# Comprehensive analysis of myocilin variants in east Indian POAG patients

**Published:** 2012-06-13

**Authors:** Deblina Banerjee, Ashima Bhattacharjee, Archisman Ponda, Abhijit Sen, Kunal Ray

**Affiliations:** 1 Molecular & Human Genetics Division, CSIR-Indian Institute of Chemical Biology, Kolkata, India; 2 Dristi Pradip, Jodhpur Park, Kolkata, India

## Abstract

**Purpose:**

Mutations in the myocilin gene (*MYOC)* account for 2%–4% of primary open angle glaucoma (POAG) cases. To date, a limited number of Indian POAG patients have been analyzed for the contribution of the gene towards the disease pathogenesis. In this study we provided a comprehensive analysis of a total of 765 eastern Indian POAG patients.

**Methods:**

In the present study 450 POAG patients and 208 ethnically matched controls were screened for the coding region of *MYOC* by using the polymerase chain reaction-direct sequencing approach; 315 POAG patients were analyzed in a previous study. Thus, our total patient cohort considering both the studies was 765. In addition, 1 kb upstream region of the gene was also examined for variants in a subset of 250 patients and 100 control samples.

**Results:**

Analysis of *MYOC* coding regions in 450 POAG patients revealed 10 novel variations including 2 frame-shift (R125SfsX158 and D273DfsX344) and 3 nonsynonymous changes (Arg33Lys, Ser331Leu, and Asp395Glu), 3 reported mutations and 4 reported polymorphisms. Gln48His, which has to date been reported only from Indian subcontinent, was identified in 4 individuals among 450 patients, taking the count to 7 individuals among 765 patients harboring the same mutation in eastern Indian cohort. Screening of 1 kb upstream region of *MYOC* in limited number of individuals yielded 5 variants but none are likely to contribute to the pathogenesis of the disease.

**Conclusions:**

*MYOC* mutations were found to account for 3% of POAG cases in our entire cohort (n=765) and Gln48His is the most common defect. This study, for the first time, reports the presence of deletion mutations in Indian patients, and represents the largest study performed in a single cohort in the Indian population.

## Introduction

Glaucoma affects approximately 70 million people and is the second leading cause of irreversible blindness worldwide [1]. Among the various subtypes, primary open-angle glaucoma (POAG) is the most common form of this disease.

POAG is a mutifactorial complex disorder where both environmental and genetic factors precipitate the disease. It has been suggested that 72% of POAG cases have some familial component [2], but on rare occasion it follows a Mendelian pattern of inheritance, including juvenile open angle glaucoma (JOAG) cases. It is characterized by progressive loss of retinal ganglion cells and atrophy of the optic nerve head, often associated with elevated intraocular pressure (IOP) caused by the reduced outflow of aqueous humor through the trabecular meshwork (TM), a meshwork of tissue lining the outflow pathway at the iridocorneal angle of the anterior chamber of the eye [3,4].

The complexity of POAG has recently been reviewed [5,6]. To date, 33 loci have been reported to be linked with POAG, but only three underlying genes have been identified: viz, myocilin (*MYOC*) [7], optineurin (*OPTN*) [8], and WD repeat domain 36 (*WDR36*) [9]. Also, heterozygous mutations in neurotrophin 4 (*NTF4*) have been reported in sporadic POAG patients [10,11], however, its involvement in POAG pathogenesis remains controversial [12,13]. In addition, recent studies show involvement of *CYP1B1* in POAG in spite of its being a candidate gene for primary congenital glaucoma (PCG) [14-16].

Myocilin was the first gene found to be linked to POAG [7]. It is located in chromosome 1 spanning a region of 17 kb. It contains three exons and is expressed as a 2.3-kb transcript with the translated product of 504 amino acids [17]. Most of the disease-causing mutations have been found in the third exon, a few in the first exon but none in the second exon. Myocilin mutants are known to cause glaucoma by gain of function [18], though the exact pathophysiology underlying *MYOC* mediated glaucoma pathogenesis is yet to be elucidated. However, it has been hypothesized that an increased level of myocilin as well as mutated form of the protein forms aggregates within the trabecular meshwork (TM) activating the unfolded protein response (UPR), finally resulting in malfunction or death of TM cells [19]. It has also been proposed that MYOC appears in the extracellular space of trabecular meshwork cells by an unconventional mechanism likely to be associated with the exosome [20].

Mutations in *MYOC* are the most widely studied and account for 2%–4% of POAG cases and around 33% of JOAG cases [7,21-24]. Two previous studies from our laboratory also showed similar contribution of the gene [25,26]. In this study, we further analyzed the involvement of myocilin variants in 450 additional POAG patients with a familial history of glaucoma or high intraocular pressure and have provided a comprehensive analysis of a total of 765 eastern Indian POAG patients.

## Methods

### Selection of study subjects

Eastern Indian POAG patients and control subjects from Kolkata, West Bengal, speaking Bengali, and belonging to the Indo-European linguistic group, were recruited from the Dristipradip Eye Clinic, Kolkata, India.

Identification of POAG patients and controls involved clinical, ocular and systemic examinations. Intraocular pressure (IOP) was measured by Goldmann applanation tonometry (Haag-Streit USA Inc., Mason, OH) followed by pachymetry (Ocuscan A; Alcon, Fort Worth, TX). The angles of anterior chamber and optic disc were assessed using a Goldmann 3-mirror gonioscope (Ocular Instrument, Bellevue, WA). The optic disc was also evaluated with a +78D lens. Automated threshold field analysis was done using the Humphrey Field Analyzer II (Carl Zeiss, Dublin, CA). The retinal nerve fiber layer (RNFL) was investigated by Scanning Laser Polarimetry (SLP) with variable corneal compensation technique (GDx-Vcc; Carl Zeiss, Dublin, CA).

POAG patients diagnosis was based on an increased intraocular pressure above 21 mmHg, significant cupping of the optic disc (>0.7) with or without peripapillary changes and the presence of clinically open angle (angle of the anterior chamber) on Gonioscopy. Though increased IOP is not an indicator of diseased status, MYOC mediated POAG pathogenesis is often associated with elevated IOP [27-29]. The suspicion of POAG was confirmed by typical reproducible visual field changes, viz. arcuate, Bjerrum, Seidel, paracentral, and annular scotoma and nasal steps and Scanning Laser Polarimetry for RNFL analysis (Nerve Fiber Indicator>30). The pre-perimetric cases were identified by RNFL analysis. The IOP, in each case was corrected for central corneal thickness (CCT). Individuals with any history of inflammation, ocular trauma (past and present), high myopia (>8 diopter) and ocular hypertension were excluded from this study. The patient pool consisted of 450 primary open angle glaucoma cases, including 44 juvenile onset cases. The age at diagnosis ranged from 15 to 88 years (mean age±SD, 57.03±12.44 years). The patients were not known to have any other eye disorder.

The controls subjects had an age >50 years (mean age±SD, 57.3±9.7 years), without any family history of glaucoma or ocular hypertension, IOP was considered to be within normal range i.e., <20 mmHg in both eyes, CCT ranging from 500 μm to 580 μm in both eyes with no visual field defect and normal Scanning Laser Polarimeter (SLP) parameters; These include a good yellowish bow-tie scan pattern, deviation map within normal limit, a good double hump pattern in conduction map, temporal-superior-nasal-inferior-temporal (TSNIT) parameters within normal limit, Nerve Fiber Indicator (Index) <30 for both eyes. Cup to disc ratio considered was <0.5 and similar in both eyes, with no defect in disc rim or margin and no sphincter hemorrhage around the disc. Individuals with high myopia (> 8 diopter), diabetes and hypertension were excluded from the control group. The study protocol adhered to the tenets of the Declaration of Helsinki and was approved by the Institutional Review Board.

### Collection of blood samples and genomic DNA preparation

Eight milliliters of peripheral blood was collected from the POAG patients and normal individuals with their written consent and transported with anticoagulant. Genomic DNA was prepared from fresh whole blood using the PAX gene blood DNA isolation kit (Qiagen, Hilden, Germany) according to the manufacturer’s protocol.

### Polymerase chain reaction and DNA sequencing

PCR reactions were performed in 20 μl reaction volumes using 80 ng of genomic DNA with Ex Prime Taq Premix (GeNet Bio, Chungnam, South Korea) to amplify exons and adjoining splice junctions using primers listed in Table 1. Briefly, each exon was amplified with an initial denaturation at 94 °C for 5 min, followed by 35 cycles of denaturation at 94 °C for 30 s, annealing at 58 °C for 30 s, and extension at 72 °C for 30 s. A final extension at 72 °C for 5 min completed the reaction. The PCR products (5 μl) were analyzed by electrophoresis in 1% agarose. Briefly, specific PCR products were purified and subjected to bi-directional sequencing with primers listed in Table 1, using an ABI 3130XL DNA sequencer with dye termination chemistry to identify any alteration of sequence.

**Table 1:** Primers used for PCR and sequencing of the *MYOC* coding region.

**Primer ID**	**Location**	**Primer sequences**	**Tm**	**Ampilcon Size (bp)**
**PCR primers**				
TIGR-1F	Exon 1	5'- GGCTGGCTCCCCAGTATATA -3'	62	762
TIGR-6R		5'- CTGCTGAACTCAGAGTCCCC -3'	64	
TIGR-8F	Exon 3	5'- TTATGGATTAAGTGGTGCTTCG -3'	54	860
TIGR-13R		5'- AGCATCTCCTTCTGCCATTG -3'	60	
**Sequencing primers**				
TIGR-3F	Exon 1	5'-AGTGGCCGATGCCAGTATAC-3'	62	
TIGR-3R		5'-CTGGTCCAAGGTCAATTGGT-3'	60	
TIGR-10F	Exon 3	5'- ATACTGCCTAGGCCACTGGA-3'	62	
TIGR-10R		5'- CAATGTCCGTGTAGCCACC -3'	62	

### Bioinformatic analysis

Novel changes were evaluated for their potential effect on the protein product using SIFT (sorting intolerant from tolerant) and Polyphen-2. The SIFT software predicts whether an amino acid substitution affects protein function. The effect of the substitution on the protein structure was assessed by PolyPhen-2.

## Results

Analysis of *MYOC* coding regions in POAG patients revealed 17 changes including 10 novel variations, 3 reported mutations and 4 reported polymorphisms. The mutations, polymorphisms and rare variants identified in POAG patients are listed in Table 2 and Table 3, respectively, with the genotype and in silico evaluations of the variants. The clinical phenotypes of the patients detected with mutations in the present study are given in Table 4.

**Table 2:** Mutations identified in *MYOC* in eastern Indian POAG patients.

						**Number of**	
**Exon**	**Nucleotide change**	**Amino acid change**	**Status**	**SIFT score**	**Polyphen prediction (Score)**	**Patients (MAF)**	**Controls (MAF)**
Exon 1	c.144G>T	Gln48His†	Reported	0.09	Possibly damaging (0.957)	7/765 (0.0046)	0/208
	c.375 Del G	R125fsX158	Novel	-	-	1/765 (0.00065)	0/208
	c.767C>T	Thr256Met†	Reported	0.15	Benign (0.229)	2/765 (0.0013)	0/208
	c.819 Del C	D273fsX344	Novel	-	-	1/765 (0.00065)	0/208
	c.992C>T	Ser331Leu	Novel	0.18	Benign (0.066)	1/765 (0.00065)	0/208
Exon 3	c.1102C>T	Gln368Stop†	Reported	-	-	3/765 (0.0019)	0/208
	c.1109C>T	Pro370Leu‡	Reported	0.03	Probably damaging (1.000)	1/765 (0.00065)	0/208
	c.1196G>A	Gly399Asp‡	Reported	0.00	Probably damaging (1.000)	1/765 (0.0013)	0/208
	c.1279G>A	Ala427Thr‡	Reported	0.02	Probably damaging (1.000)	2/765 (0.0013)	0/208

From a cohort of 765 POAG patients, 450 DNA samples were screened for the coding region of *MYOC* in the present study; the remaining 315 have been analyzed before [25]. Unrelated control subjects (n=208) were enrolled from the same ethnic background. MAF: Minor Allele Frequency. Genotypes for all mutations are heterozygous except for Gly399Asp. SIFT score predicts phenotypic effect; <0.05 predicted to be deleterious, ≥0.05 tolerated. SIFT score cannot be calculated for the nonsense/in-del variants. The effect of the substitution on the protein structure was assessed by PolyPhen-2. † Present study and previously published [25], ‡ Previously published [25].

**Table 3:** Polymorphic and rare variants in *MYOC* in eastern Indian POAG cohort.

				**Number of**	
**Location**	**Nucleotide change**	**Amino acid change**	**Status**	**Patients (MAF)**	**Controls (MAF)**
Promoter	−83G>A*	-	Reported	150/ 250 (0.33)	67/100 (0.38)
	−159T>C*	-	Novel	12/250 (0.024)	4/100 (0.02)
	−190T>C	-	Reported	1/250 (0.002)	0/100
	−241G>A*	-	Novel	18/250 (0.036)	6/100 (0.03)
	−320C>T*	-	Novel	24/250 (0.048)	10/100 (0.05)
Exon 1	c.98G>A	Arg33Lys	Novel	1/765 (0.00065)	0/208
	c.227G>A*	Arg76Lys†	Reported	459/765 (0.33)	139/208 (0.38)
	c.309 C>T	Thr103Thr	Novel	1/765 (0.00065)	0/208
	c.372 G>C	Leu124Leu	Novel	1/765 (0.00065)	0/208
	c.419 C>G	Thr140Ser	Novel	0/208	1/765 (0.00065)
Exon 3	c.816 A>T	Arg272Arg	Novel	1/765 (0.00065)	0/208
	c.855 G>T	Thr285Thr‡	Reported	1/765 (0.00065)	0/208
	c.952C>T	Leu318Leu‡	Reported	1/765 (0.00065)	0/208
	c.1041T>C	Tyr347Tyr†	Reported	18/765 (0.012)	1/208 (0.002)
	c.1053C>T	Thr351Thr	Reported	1/765 (0.00065)	0/208
	c.1058C>T	Thr353Ile‡	Reported	1/765 (0.00065)	0/208
	c.1182C>T	Thr394Thr‡	Reported	1/765 (0.00065)	0/208
	c.1185T>G	Asp395Glu	Novel	1/765 (0.00065)	0/208
	c.1239C>T	Leu413Leu	Novel	1/765 (0.00065)	0/208
	c.1278C>T	Val426Val	Novel	1/765 (0.00065)	0/208

From a cohort of 765 POAG patients, 450 DNA samples were screened for the coding region of *MYOC* in the present study; the remaining 315 have been analyzed before [25]. Unrelated control subjects (n=208) were enrolled from the same ethnic background. One kb upstream region of the gene was also examined for variants in 250 patients and 100 control samples. MAF: Minor Allele Frequency. † Present study and previously published [25], ‡ Previously published [25].

**Table 4:** Clinical data of POAG patients harboring mutations in *MYOC*.

**Patient ID (age at diagnosis in years)**	**Mutation**	**Subtype**	**IOP (mm of Hg; RE, LE)**	**Untreated IOP (mm of Hg; RE, LE)**	**C:D ratio (RE, LE)**	**Visual field changes**	**Nerve Fiber Index (NFI; RE, LE)**	**Prescribed medicine**	**Other systemic disorders**
GL1240 (64)	Arg33Lys	POAG (Familial)	20, 21	20, 21 (post-operative)	0.8, 0.8	Double arcuate defect (BE)	N/A	Timolol	Cataract
GL634 (64)	Gln48His	POAG (Sporadic)	11, 12	11, 12 (post-operative)	0.8, 0.8	Vision lost (RE), Gross constriction (LE)	N/A	Brimonidine	Hypertension
GL925 (56)	Gln48His	POAG (Familial)	18,17	23,20.4	0.7, 0.6	Superior Zone depression (RE), Superior & nasal defect (LE)	63, 46	Brimonidine	N/A
GL937 (58)	Gln48His	POAG (Familial)	30, 25	30, 25	N/A	Sup arcuate defect & inferior zone depression (RE) Double arcuate zone depression (LE)	31, 42	Latanoprost	Diabetes Mellitus, Hypertension
GL1000 (25)	Gln48His	JOAG (Familial)	13.7, 37.7	13.7, 37.7	0.4, 0.9	Minimum change in visual field (RE) Vision is PL positive (LE)	27, 27	Brimonidine +Timolol, Bimatoprost	N/A
GL883 (67)	R125SfsX158	POAG (Familial)	25, 21	32.5,27	0.9, 0.9	Superior hemifield defect (RE) Superior arcuate defect (LE)	N/A	Brimonidine +Timolol	Cataract
GL1145 (43)	Thr256Met	POAG (Sporadic)	21, 45	21, 45	N/A	Enlarged blind spot (RE) Double arcuate defect (LE)	N/A	Latanoprost, Dorzolamide	Coronary artery disease
GL1129 (64)	D273DfsX344	POAG (Familial)	22.9, 37.9	22.9, 37.9	N/A	Superior arcuate defect, (RE) Superior arcuate defect (LE) Enlarged blind spot (BE)	N/A	Latanoprost, Dorzolamide	Cataract
GL1208 (61)	Ser331Leu	POAG (Familial)	22, 21	22, 21	0.5, 0.5	Generalized depression (BE)	22, 27	Timolol, Betaxolol	Cataract, Bypass surgery, Diabetes Mellitus, Hypertension
GL796 (56)	Asp395Glu	POAG (Sporadic)	24, 25	24, 25	N/A	Generalized depression (BE)	N/A	N/A	Cataract
GL1104 (49)	Asp395Glu	POAG (Familial)	21.9, 20.4	27.3,25.5	0.7, 0.6	Enlarged blind spot (BE)	42, 48	Levobunolol	N/A
GL1117 (55)	Gln368Stop	POAG (Sporadic)	­­41.3, 47	41.3, 47	N/A	Generalized depression with double arcuate defect (RE) Superior arcuate zone depression (LE)	17, 20	Dorzolamide	N/A

Abbreviations used: RE: right eye and LE: left eye; IOP: Intra ocular pressure; C:D ratio: cup to disc ratio; PO: post operative; BE: both eye, PL: Perception of light.

Among the novel variants identified, c.98G>A, Arg33Lys was identified in a 64-year-old glaucoma patient with a positive family history of glaucoma (Table 4). Due to a lack of family members we could not ascertain the pathogenic effect of this variation.

**Figure 1:**
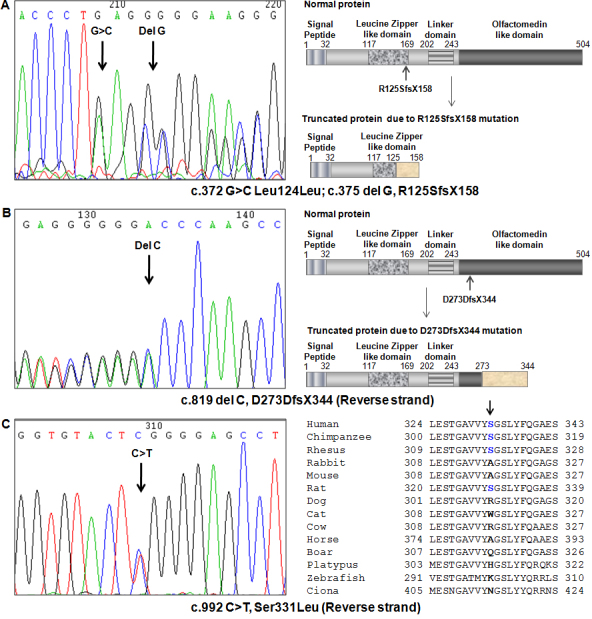
Novel changes identified in myocilin in POAG patients. All the changes were identified in the heterozygous condition. The mutated base is indicated with an arrowhead in the chromatograms. **A**: The chromatogram on the left demonstrates location of a synonymous variant (c.372 G>C, Leu124Leu) and a deletion mutation (c.375, del G, R125SfsX158). On the right, the cartoons show all the known domains of normal MYOC and the truncated protein resulting from the deletion, including aberrant 33 amino acids at the COOH-terminal end. **B**: The chromatogram on the left demonstrates location of a deletion mutation (c.819, del C, D273DfsX344). On the right, the cartoons demonstrate the known domains of normal MYOC and the truncated protein resulting from the deletion, including aberrant 71 amino acids at the COOH-terminal end. **C**: The chromatogram on the left demonstrates location of a nonsynonymous variant (c.992 C>T, Ser331Leu). On the right, conservation status of the residue (indicated by arrowhead) is shown in homologous protein in other species.

A frameshift mutation c.375 Del G, R125SfsX158 was identified in *MYOC* exon 1 (Figure 1A). The patient (GL883) was detected with POAG at 67 years of age and a positive family history (Table 4) with both the elder brother and sister being affected. Clinical examination revealed that she has severe visual field defect with high cup disc ratio in both eyes (RE>LE). Analysis of the blood samples from her son and daughter showed the presence of the probable disease causing mutation in her 42-year-old daughter. However, there were no early signs of glaucoma. Another novel frameshift mutation c.819 Del C, D273DfsX344 in exon 3 (Figure 1B) was identified in a 64-year-old POAG patient (GL1129) with positive family history (Table 4). Apart from the proband, his 27-year-old daughter harbored the same nucleotide change and had an IOP of 23 mmHg and 21 mmHg with a cup:disc ratio of 0.3 and 0.2 in her right and left eyes, respectively. She was phenotypically normal. However, she was advised to be under constant supervision at regular intervals. By conceptual translation of the transcript both these frameshift mutations are predicted to result in early truncation of *MYOC*.

A c.992C>T, Ser331Leu variant (Figure 1C) was identified in a 61-year-old male patient (GL1208) with a positive family history of glaucoma (Table 4), with his late father being affected with severe glaucoma phenotype. Analysis of blood samples from his family members showed that the absence of the variation in his son and daughter. The variation involves replacement of an evolutionarily conserved amino acid by a nonconservative change of the residue, which was not detected in the controls. A previous study from south India identified a change to threonine at the same position [30].

The novel variation c.1185T>G, Asp395Glu in *MYOC* exon 3 was identified in two unrelated adult onset glaucoma cases (Table 4), both of whom were heterozygous for the change. The change is assessed to be benign by in silico analysis.

Among the reported mutations, c.144G>T, Gln48His, which has only been reported from the Indian subcontinent to date [23,26,31,32], was identified in 4 of the 450 POAG patients analyzed in the current study. Thus, to date, 7 (4 POAG & 3 JOAG) out of 765 patients in the eastern Indian cohort were found to harbor the same mutation. All the identified individuals were high tension glaucoma patients, which points toward the pathogenic effect of the mutation resulting in a severe phenotype. A Triton-X-100 assay [33], done using recombinant myocilin containing the variant residue (His) shows aggregation, confirming the deleterious effect of the mutation (unpublished). Genotyping done with the microsattelite markers DIS2815 and DIS2790 and −83G/A single nucleotide polymorphism (SNP) in *MYOC* suggests that the mutation might have originated multiple times in different haplotype backgrounds.

In the course of the study, c.767C>T, Thr256Met was identified in two POAG patients in heterozygous state. Interestingly, both these individuals had no family history of glaucoma. The age at diagnosis was similar (43 years and 46 years) with enlargement of blind spots. This similarity in disease phenotype points toward a potentially common underlying pathophysiology of the mutation. Although Polyphen analysis predicts this variant to be a benign polymorphism, the threonine residue is found to be conserved across different species. The Thr256Met change has also been reported in a single patient from a study of normal tension glaucoma [34]. However, that study did not provide a detailed clinical description of the patient for further analysis. Unlike simple Mendelian disorders, glaucoma is a complex disease, and it is quite possible that the variants predicted to be benign might act as modifiers under a permissible environment, and thus might show varying levels of penetrance in the family.

A c.1102C>T, Gln368Stop mutation was identified in a patient (GL1117) with high intraocular pressure of 41.3 mmHg (RE) and 47 mmHg (LE). Presence of this mutation has previously been reported from our laboratory in 2 unrelated families among which one family showed incomplete penetrance [25]. The family members of GL1117 were not available for examination. This mutation is common among Caucasians [35] with a prevalence of 29.3% in the world.

In addition, previous studies from the laboratory identified three more mutations Pro370Leu, Gly399Asp, and Ala427Thr (Table 2). Thus, comprehensive analysis of *MYOC* coding regions in a cohort of 765 POAG patients from eastern India, including previous studies [25,26], led to identification of 12 mutations in 23 patients.

### Synonymous changes in the *MYOC* coding region

In addition to other variants, 10 synonymous changes were identified in heterozygous condition only in patients and not in controls (Table 3). Among these variants, c.1239C>T (Leu413Leu) was predicted to undergo a change in the *MYOC* mRNA structure with a variation in structural energy (∆G) of 15kJ (from −486.66kJ to −471.00kJ) that might affect the protein stability.

### Variants identified in the *MYOC* promoter region

The promoter region of *MYOC* has multiple steroid hormone response elements and regulatory motifs [36]. Polymorphisms in the *MYOC* upstream region could influence regulation of its expression and their possible consequences on predisposition to POAG. Analysis of 1Kb of the proximal promoter of *MYOC* in 250 POAG patients and 100 controls led to the identification of 5 nucleotide variants which are listed in Table 3. Among these variants −83G>A is widely reported but −159T>C, −241G>A, and −320C>T represent novel polymorphisms. No significant difference was observed in the distribution of alleles among the patients and controls. The nucleotide variant −190T>C, i.e the 4th base of a consensus putative negative glucocorticoid response element (nGRE) was identified in a single POAG patient. However, this change has been previously identified in controls in other populations [37,38].

## Discussion

The study on the eastern Indian POAG cohort (n=765) from this group led to identification of 12 mutations (5 novel and 7 reported) in *MYOC* of 23 patients (3%) which is consistent with the world average (2%–4%). The mutations include 9 nonsynonymous changes, 2 deletions and 1 nonsense mutation, and most of the mutations (9/12) are located in exon 3 which codes for the olfactomedin domain, reported to be important for the functionality of the protein [39]. No significant change was identified in the promoter region of *MYOC*.

To date, involvement of *MYOC* in POAG has been examined mostly in eastern and southern parts of India only. The report by Chakrabarti et al. [31] in 2005 included 13.5% patients from western India and 23.5% patients from northern India in a cohort of 251 patients, but no POAG causing mutation in *MYOC* was identified in these two groups. A complete list of mutations in the *MYOC* gene identified in the Indian population is listed in the Indian Genetic Disease Database [40]. Among the identified genetic defects, Gln48His is the most prevalent mutation detected in the Indian population. Interestingly, this mutation has not been reported from any other part of the globe.

As discussed in the results section, in addition to nonsynonymous changes, two frame shift mutations, viz. R125SfsX158 and D273DfsX344, resulting from single base deletions were identified in patients having a positive family history of glaucoma. The mutations resulted in early termination and formation of truncated proteins lacking the COOH-terminal olfactomedin domain of myocilin, a functionally important region. To date, small deletion accounts for 2.3% of the mutations identified in myocilin and all of them have been marked as glaucoma causing mutations [41].

Studies done on *MYOC* suggest that haploinsufficiency is not a critical mechanism for POAG in individuals with mutations in the gene. There has been a lack of discernible phenotype in both *Myoc*-heterozygous and *Myoc* null mice [18]. Also, absence of POAG in carriers with *MYOC* homozygous mutations, in contrast to disease phenotype in carriers of heterozygous mutations [42], points toward the fact that disease-causing mutations in humans likely act by gain of function. The COOH-terminal domain of myocilin is of functional importance. Calpain binds in the olfactomedin domain of myocilin [43] and proteolytic cleavage of myocilin occurs between amino acids Arg226 and Ile227 [39].

Conceptual translation of the frameshift mutation R125SfsX158 in exon 1 produces a mutated protein of 158 amino acids (including aberrant 33 amino acids at the COOH-terminal end). Similarly, D273DfsX344 in exon 3 would produce a 344 amino acid protein with 71 aberrant amino acids. These heterozygous forms of the mutated protein interferes with the proper functioning of the native myocilin by increasing the secretion of the mutated form and reducing the secretion of the extracellular processed myocilin, thus causing POAG by dominant gain of function mechanism [44]. In this context, others [45-47] have also demonstrated that mutation or absence of the olfactomedin domain of myocilin results in a misfolded protein product, which suppresses the proper secretion of the protein. This misfolded protein accumulates in the endoplasmic reticulum [48,49], causing ER stress and subsequently resulting in cell death [48-50]. This proves that mutations in the olfactomedin domain are expected to give rise to possible pathogenic glaucoma phenotypes, and explains preponderance of disease causing mutations in *MYOC* exon 3 coding this domain.

This study, according to our knowledge, is the largest study to date performed in a single cohort in the Indian population. The involvement of the gene in 3% of the population is similar to what is reported in the world literature. The identification of *MYOC* mutations in probands and preclinical diagnosis of the individuals at risk will help clinicians in better management of the disease.
